# Pepper Novel Pseudo Response Regulator Protein CaPRR2 Modulates Drought and High Salt Tolerance

**DOI:** 10.3389/fpls.2021.736421

**Published:** 2021-10-20

**Authors:** Junsub Lim, Chae Woo Lim, Sung Chul Lee

**Affiliations:** Department of Life Science (BK21 Program), Chung-Ang University, Seoul, South Korea

**Keywords:** abscisic acid, drought stress, high salinity, pepper, stomata

## Abstract

Plants modify their internal states to adapt to environmental stresses. Under environmental stress conditions, plants restrict their growth and development and activate defense responses. Abscisic acid (ABA) is a major phytohormone that plays a crucial role in the osmotic stress response. In osmotic stress adaptation, plants regulate stomatal closure, osmoprotectant production, and gene expression. Here, we isolated *CaPRR2* – encoding a pseudo response regulator protein – from the leaves of pepper plants (*Capsicum annuum*). After exposure to ABA and environmental stresses, such as drought and salt stresses, *CaPRR2* expression in pepper leaves was significantly altered. Under drought and salt stress conditions, *CaPRR2*-silenced pepper plants exhibited enhanced osmotic stress tolerance, characterized by an enhanced ABA-induced stomatal closing and high MDA and proline contents, compared to the control pepper plants. Taken together, our data indicate that CaPRR2 negatively regulates osmotic stress tolerance.

## Introduction

Plants are sessile organisms; hence, they develop stress response mechanisms to adapt to various environmental stresses. Osmotic stresses, such as drought and salinity, disrupt homeostasis and cause functional and structural damage to proteins, resulting in fatal injury to cells (Wang et al., 2003; Golldack et al., 2014; Zhao et al., 2021). Plants enhance their osmotic tolerance by adjusting physiological and molecular processes, such as osmotic adjustment and antioxidant production, in response to stress conditions (Ma et al., 2020). During drought stress, plants regulate water status and alter gene expression *via* osmotic stress response signaling (Osakabe et al., 2011). Abscisic acid (ABA) is a key phytohormone that regulates osmotic stress responses, including physiological and molecular changes (Fujita et al., 2011). Under high-salt stress conditions, plants perceive salt and import sodium. Perception leads to early signaling responses, such as the activation of K^+^ transport and Ca^2+^ signaling as well as the induction of reactive oxygen species (ROS). Additionally, plants regulate hormone levels and gene expression. Consequently, adaptive responses occur, and plants are able to survive under osmotic stress conditions (van Zelm et al., 2020). Osmotic adjustment through ions occurs *via* the alteration of the K^+^/Na^+^ balance in roots and shoots (Almeida et al., 2017). Moreover, osmotic adjustment *via* organic solutes occurs through the synthesis and accumulation of organic solutes in the cytoplasm (Quero et al., 2014). Plants alleviate ion toxicity by reestablishing cell turgor and driving the gradient for water uptake to minimize damage. However, these processes inhibit plant growth and development.

Two-component signaling systems (TCSs) function in various signal transduction pathways in many prokaryotes and a few eukaryotes, which simply consist of two elements: histidine protein kinase and a response regulator protein (Stock et al., 2000; Satbhai et al., 2011). In plant, several components of TCSs are found to be involved in plant hormone, stress, and light signaling (Hwang et al., 2002; Mizuno, 2005). Among them, the pseudo-response regulators (PRR), one of the three types of response regulator proteins, contain an atypical receiver domain; in Arabidopsis, nine PRRs does not have the phosphor-accepting Aspartic acid residue which is conserved in receiver domain of response regulator protein (Hwang et al., 2002; Mizuno, 2005). Within this type genes, PRR2 have been proven to have no phospho-accepting activity in its receiver domain (Makino et al., 2002). PRR2 contains a Myb-like DNA-binding domain (also referred to GARP domain) at the C-terminus, together with atypical receiver domain at the N-terminus (Hwang et al., 2002). Unlike other PRRs involved in the circadian clock mechanism (Mizuno and Nakamichi, 2005), biological function of PRR2 remains largely unknown. Recent studies have revealed that in Arabidopsis PRR2 functions as a positive regulator of plant immunity by enhancing salicylic acid (SA) biosynthesis and SA signaling responses (Cheval et al., 2017). This *PRR2* transcript expression increased in response to inoculation with *Pseudomonas syringae* and SA treatment. Moreover, *prr2* knockdown and overexpression mutants exhibited altered susceptibility to *P. syringae*, induction of defense marker genes, and altered PR1 protein levels and SA content (Cheval et al., 2017). Arabidopsis PRR2 has been isolated an interacting partner of calmodulin-like protein 9 (CML9; Perochon et al., 2010). *CML9* gene, rapidly induced by both biotic and abiotic stress such as cold and drought, is associated with salt tolerance through modulation of ABA-mediating pathway (Magnan et al., 2008), providing the potential for its interacting partner PRR2 to be involved in response to abiotic stress. However, the precise function of PRR2 still remains unknown.

In the present study, we isolated *CaPRR2* (*Capsicum annuum* Pseudo-Response Regulator 2), a homolog of Arabidopsis PRR2, and investigated its functional involvement in pepper plants in response to drought and salt stress. The CaPRR2 protein contains an MYB domain at its C-terminus. *CaPRR2* transcripts were induced or repressed in response to various stress treatments, and CaPRR2 was localized in the nucleus by the MYB domain. We used loss-of-function genetic studies to examine the functional roles of CaPRR2 in response to exogenous ABA and osmotic stress treatments. *CaPRR2*-silenced pepper plants exhibited enhanced ABA sensitivity and abiotic stress-tolerant phenotypes. Moreover, under drought and high salinity conditions, *CaPRR2* knockdown pepper plants showed high expression levels of osmotic response genes. Taken together, our data indicate that CaPRR2 negatively regulates osmotic stress resistance.

## Materials and Methods

### Plant Materials and Growth Conditions

Korean red pepper (*C. annuum* L., cv. Nockwang) and tobacco (*Nicotiana benthamiana*) seeds were sown in loam, sand, and a compost mix of soil (vermiculite, perlite, and, peat moss, 2:3:5, v/v/v; 1:1:1, v/v/v). Plants were placed in growth room at 24±1°C with 60% relative moisture under white fluorescent light (130μmol photons·m^−2^·s^−1^) with a 16-h light/8-h dark cycle.

### Virus-Induced Gene Silencing

To generate the *CaPRR2*-silenced pepper plants, a tobacco rattle virus (TRV)-based virus-induced gene silencing (VIGS) technique was employed, as described by Lim et al. (2018). Using the VIGS tool,^1^ two 300-bp fragments of the *CaPRR2* cDNA, *CaPRR2* N1 (1,052–1,351), and *CaPRR2* N2 (1,361–1,660) were designed to avoid off-target of silencing; each region was subsequently amplified by PCR using the primers *Xba*I-CaPRR2 N1 (5ʹ-TCTAGATGAAAGTAGAAGGCCTGACAA-3ʹ) and *Xho*I-CaPRR2 N1 (5ʹ-CTCGAGTCTCGGGTGGTTGCCAT-3ʹ) or *Xba*I-CaPRR2 N2 (5ʹ-TCTAGAGGAATCCTCACTCTGGACTGTAT-3ʹ) and *Xho*I-CaPRR2 N2 (5ʹ-CTCGAG GAGAACCGTTGATGCGTG-3ʹ). *Agrobacterium tumefaciens* strain GV3101 carrying pTRV1 and pTRV2:*CaPRR2* N1, pTRV2:*CaPRR2* N2, or pTRV2:00 as a negative control was co-infiltrated into the fully expanded cotyledons of pepper plants (OD_600_=0.2 for each construct). Infected plants were placed in a growth room and maintained under the growth conditions described above for growth and spread of the virus.

### RNA Isolation and Quantitative Reverse Transcription-Polymerase Chain Reaction

Total RNA isolation and reverse transcription-polymerase chain reaction (RT-PCR) analyses were performed as described previously with some modifications (Lim and Lee, 2016; Lim et al., 2018). Pepper leaves at six-leaf stage were treated with ABA (100μM), H_2_O_2_ (100mM), mannitol (600mM), low temperature (10°C), NaCl (200mM), or drought stresses. cDNA was synthesized using harvested pepper leaves by a Transcript First Strand cDNA Synthesis kit (Roche, Indianapolis, IN, United States). The cDNA synthesized for quantitative real-time polymerase chain reaction (qRT-PCR) assay was amplified in a CFX96 Touch™ Real-Time PCR detection system (Bio-Rad, Hercules, CA, United States) using iQ™SYBR Green Supermix and specific primers (Supplementary Table S1). The relative expression level of each gene was calculated using the ∆∆*C*t method as previously described (Livak and Schmittgen, 2001). The pepper *Actin1* (*CaACT1*) gene was used for normalization (Lim and Lee, 2016).

### *In silico* Analysis

Using the deduced amino acid sequence of *CaPRR2 gene* as query, its isoelectric point and a molecular weight were calculated in web tool Expasy Compute pI/MW.^2^ Protein sequences of *CaPRR2*-homologous genes from other plant species were obtained through BLASTP search and were used for multiple sequence alignment analysis using web tool Clustal Omega.^3^ The phylogenetic tree analysis was conducted using MEGA software (version 7.0) with the neighbor-joining method.

### Subcellular Localization

To determine the subcellular location of CaPRR2, the full-length coding sequence (1–557) and fragments of *CaPRR2* (1–151, 152–291, 292–451, 452–557, and 332–451bp) without the stop codon were inserted into the p326GFP vector. The GV3101 strain of *Agrobacterium tumefaciens* containing each construct was mixed with strain p19 (1:1 ratio; OD_600_=0.5) and co-inoculated into fully expanded leaves of 5-week-old *N. benthamiana*. After 2days, the green fluorescent protein (GFP) signals were detected under a confocal microscope (510 UV/Vis Meta; Zeiss) equipped with LSM Image Browser software.

### Drought Treatment

Three-week-old control and *CaPRR2*-silenced pepper plants were subjected to drought stress by withholding water for 14days. The survival rate was measured by counting the plant number resumed their growth 2days after re-watering. To determine the water loss, the first and second leaves were detached from both pepper plants. The fresh weights of the detached leaves were measured hourly. For the analysis of the relative water content (RWC), the first leaves were detached from 3-week-old pepper plants of each line, and the fresh weight (FW) was measured. The leaves were incubated under turgid conditions for 8h, and the turgid weight (TW) was measured. The dry weight (DW) was measured after drying in a 60°C dry oven for 4days. The RWC of each plant line was calculated using the formula [RWC=(FW−DW)/(TW−DW)×100]. For qRT-PCR analysis, 3-week-old TRV2:*CaPRR2* and TRV2:00 plants were carefully removed from the soil and subjected to drought stress. After 6h, the first and second leaves of each plant line were harvested, and total RNA was isolated. The experiments were repeated three times.

### Thermal Imaging and Stomatal Aperture Bioassay

Measurements of the leaf temperature and stomatal pore size were performed as previously described (Lim and Lee, 2016). Six-leaf stage pepper plants were used for the thermal image analysis. Thermal images were taken before and after treatment with 0 and 100μM ABA for 6h using an infrared camera (FLIR system; T420), and leaf temperature was measured with FLIR Tools + version 5.2 software.

TRV2:*CaPRR2* and TRV2:00 leaf peels of the first and second leaves were floated on stomatal opening solution (SOS: 10mM CaCl_2_, 10mM MES-KOH, pH 6.15, and 50mM KCl) under light conditions for 2.5h. After 2.5h, the leaf peels were transferred to fresh SOS containing 0 and 20μM ABA and incubated for an additional 2.5h. For measurement of the stomatal apertures, 100 stomata per sample were observed under a Nikon Eclipse 80i microscope. Each experiment was performed independently three times.

### Salt Treatment

Three-week-old *CaPRR2*-silenced and control pepper plants were hydroponically subjected to salt stress using water containing 200mM NaCl. The survival rate was calculated by counting the number of plants 4days after salt stress treatment. To determine the chlorophyll content, various concentrations of NaCl solution were applied to the leaf discs of TRV2:*CaPRR2* and TRV2:00 plants. Five days after salt stress treatment, the leaf disc chlorophyll content was measured spectrophotometrically, as described previously (Lim et al., 2015). For qRT-PCR analysis, 3-week-old TRV2:*CaPRR2* and TRV2:00 plants were hydroponically subjected to salt stress using water containing 200mM NaCl. After 6h, the first and second leaves of each plant line were harvested, and total RNA was isolated. All experiments were repeated three times.

### Measurement of Proline and Malondialdehyde Contents

Three-week-old *CaPRR2*-silenced and control pepper plants were subjected to salt stress by hydroponically incubating them to various concentrations of NaCl solution. Proline contents of harvested leaf samples were determined by a ninhydrin-based colorimetric assay at 520nm as described by Abrahám et al. (2010). Malondialdehyde (MDA) contents were measured by thiobarbituric acid (TBA) assay as previously described (Heath and Packer, 1968; Lim et al., 2015).

### Statistical Analyses

Statistically significant differences between genotypes were determined using Student’s *t* test. Results were considered significant at *p*<0.05.

## Results

### Isolation of the *CaPRR2* Gene

**Figure 1:**
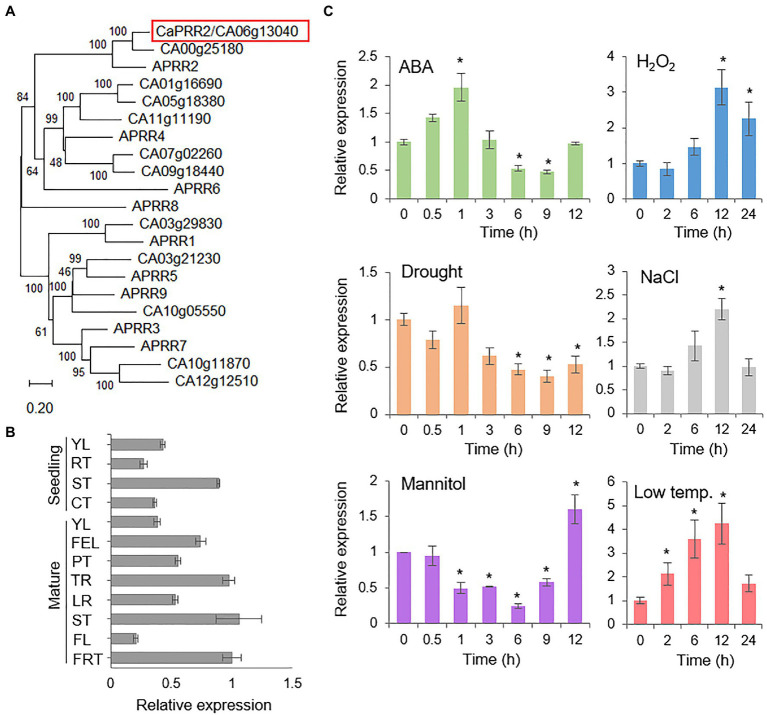
Expression patterns of the *CaPRR2* gene. **(A)** Phylogenetic tree analysis of the CaPRR2 protein and its homologous proteins in *Arabidopsis thaliana*. Using a BLAST search with CaPRR2 as query, protein sequences of *A. thaliana* were retrieved. For multiple sequence alignment, the web tool Clustal Omega^3^ was used with the default settings. The phylogenetic tree was generated using MEGA X software on the basis of multiple alignments of CaPRR2 and its homologous proteins from *A. thaliana* in ClustalW2. **(B)** Tissue-specific expression of *CaPRR2* in various pepper plant tissues at the seedling and mature stages. YL, young leaf; RT, root; ST, stem; CT, cotyledon; FEL, fully expanded leaf; PT, petiole; TR, tap root; LR, lateral root; FL, flower; FRT, fruit. **(C)** Expression patterns of *CaPRR2* in the leaves of pepper plants exposed to abscisic acid (ABA; 100μM), drought, H_2_O_2_ (100mM), mannitol (600mM), NaCl (200mM), or low temperature (10°C). The pepper *Actin1* (*CaACT1*) gene was used as an internal control. Data represent the mean±SE of three independent experiments; asterisks indicate significant difference compared with the untreated control (0h; Student’s test; *p*<0.05).

To isolate PRR genes from pepper plants, we conducted BLASTP search using amino acid sequences of nine Arabidopsis *PRR* (APRR) genes as queries and found 12 gene loci for putative *CaPRR* in pepper plants. To determine their genetic relation, we performed a phylogenetic tree analysis with the deduced amino acid sequences of 12 *CaPRR* genes and 9 *APRR* genes and found that these genes were simply clustered into two groups (Figure 1A). There were one or two candidate genes of PRR homolog, corresponding to APRR1, APRR2, APRR3, APRR5, APRR7, and APRR9, in the pepper genome. Of the APRRs, it has been suggested that APRR2 may be associated with plant responses to abiotic stress, such as salt and drought, based on the interaction with CML9 (Perochon et al., 2010). Hence, we selected CA06g13040 as APRR2 homolog for further study and named CaPRR2; in protein sequence, CA06g13040 shares 48.2% identity/59.5 similarity with APRR2, but CA00g25810 shares 44% identity/55.7% similarity. The *CaPRR2* gene consists of a 1,674-bp open reading frame, encoding 557 amino acid residues with an isoelectric point of 6.17 and a molecular weight of 61.81kDa. Multiple sequence alignment analysis revealed high amino acid sequence identity (48.2–85.1%) and similarity (59.4–89.3%) between CaPRR2 and other plant PRR2 proteins (Supplementary Figure S1). CaPRR2 contains a highly conserved cheY-homologous receiver domain and an MYB-like DNA-binding domain.

To investigate the organ-specific expression of *CaPRR2*, we examined *CaPRR2* transcript levels in various pepper plant tissues using quantitative RT-PCR analysis. At the seedling and mature stages, *CaPRR2* was strongly expressed in young leaf and stem, and compared to these organs, the others, such as root, flower, and green fruit, had <50% expression levels (Figure 1B). To investigate whether *CaPRR2* is associated with the environmental stress response, we examined the induction of *CaPRR2* transcripts after exposure to ABA, drought, H_2_O_2_, mannitol, NaCl, and low temperature (Figure 1C). The *CaPRR2* transcripts tended to decrease 3h after exposure to ABA, drought, and mannitol by low than 50%. In response to H_2_O_2_ and NaCl treatment, the *CaPRR2* expression levels were peaked (1.6 to 2-fold) at 12h. Low-temperature exposure led to gradual induction of *CaPRR2*, particularly at 12h, with 5-fold increase. These results suggest that *CaPRR2* may be involved in the abiotic stress signaling.

### Subcellular Localization of the CaPRR2 Protein by the MYB Domain

**Figure 2:**
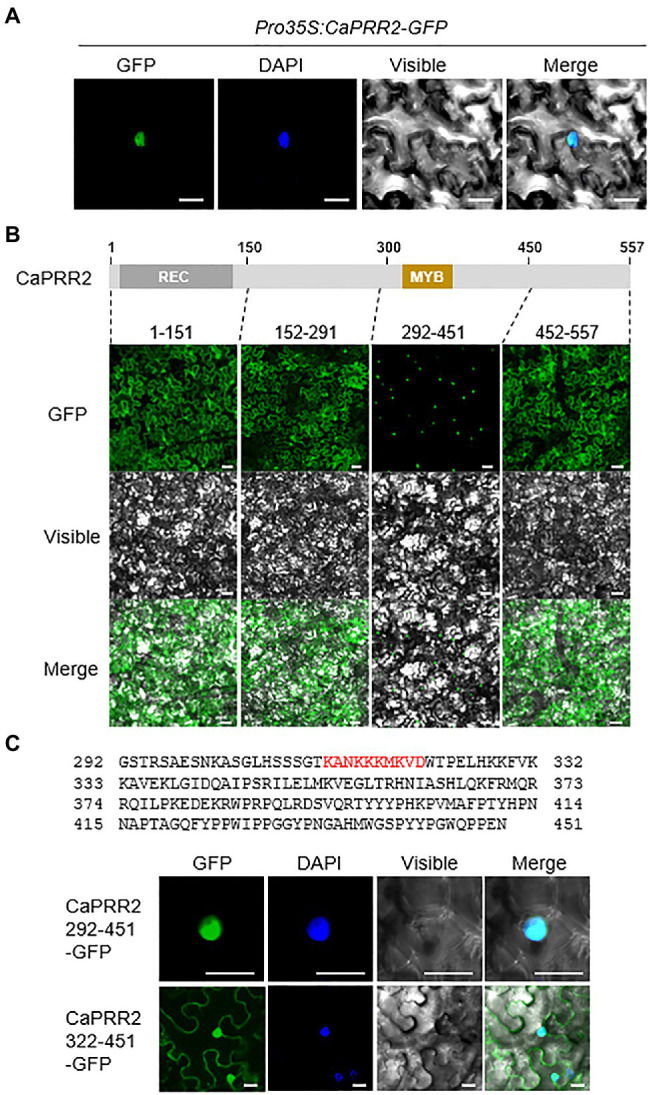
Nuclear localization of CaPRR2 by the MYB domain. **(A)** Nuclear localization of the CaPRR2-green fluorescent protein (GFP) fusion proteins in the epidermal cells of *Nicotiana benthamiana*. **(B)** Subcellular localization of the fractionated CaPRR2s in the epidermal cells of *N. benthamiana*. **(C)** Subcellular localization of the fractionated CaPRR2s containing the MYB domain in the epidermal cells of *N. benthamiana*. Cells were stained with 4ʹ,6-diamidino-2-phenylindole (DAPI) for nuclear localization. White bar=10μm.

To investigate the subcellular localization of CaPRR2 in plant cells, the GFP reporter gene was fused to the C-terminal region of *CaPRR2* under the control of the 35S promoter (*Pro35S:CaPRR2-GFP*), and the GFP-fused proteins were transiently expressed in the epidermal cells of *N. benthamiana*. Using the nuclear marker, diamidino-2-phenylindole, we showed that the GFP signals were localized in the nucleus (Figure 2A). To investigate the domain that determines CaPRR2 localization in the nucleus, we fractionated CaPRR2 and observed the localization of the resulting constructs (Figure 2B). The deletion construct carrying the MYB domain (292–451 amino acid residues) was localized only in the nucleus. To verify whether the MYB domain is associated with CaPRR2 localization in the nucleus, we fractionated this construct, except for the MYB domain (322–451 amino acid residues; Figure 2C). The resulting construct was localized in the nucleus and the cytoplasm. These results suggest that CaPRR2 is localized in the nucleus by the MYB domain and functions in the nucleus.

### Enhanced Drought Tolerance of *CaPRR2*-Silenced Pepper Plants

**Figure 3:**
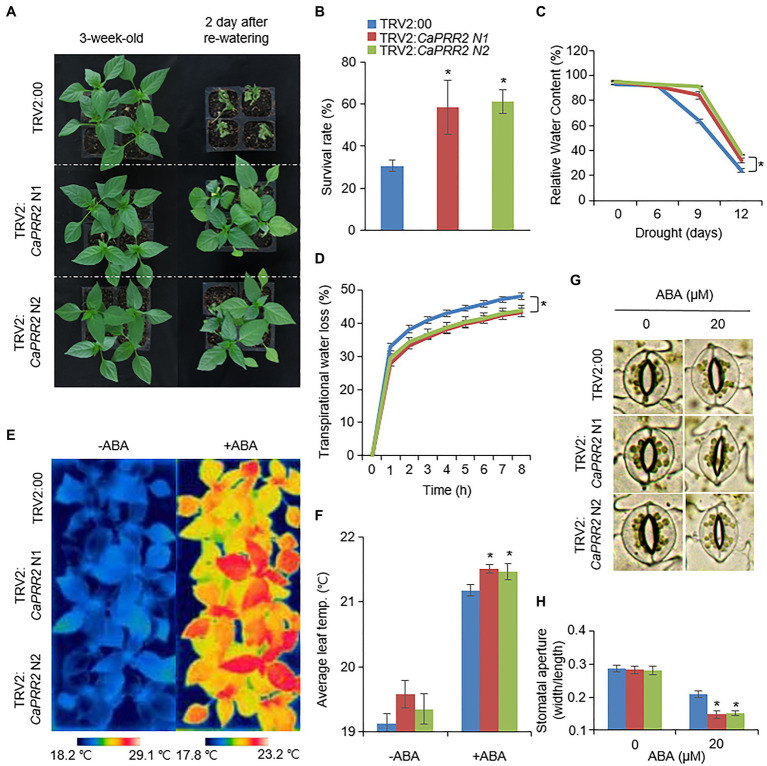
Enhanced drought tolerance of *CaPRR2*-silenced pepper plants. **(A)** Drought sensitivity of *CaPRR2*-silenced (TRV2:*CaPRR2*) and control (TRV2:00) pepper plants. Three-week-old plants of each line were exposed to drought stress by withholding watering for 14days. After re-watering for 2days, the representative images were taken. **(B)** Survival rates of pepper plants after re-watering. Data represent the mean±SE of three biological replicates, each evaluating 20 plants. **(C)** Relative water contents of TRV2:*CaPRR2* and TRV2:00 plants exposed to drought stress. **(D)** Transpirational water loss from the leaves of TRV2:*CaPRR2* and TRV2:00 plants. Leaves were detached and the fresh weights of each line were measured at the indicated time points. **(E,F)** Enhanced leaf temperatures of *CaPRR2*-silenced (TRV2:*CaPRR2*) and control (TRV2:00) pepper plants in response to abscisic acid (ABA) treatment. Thermal images of *CaPRR2*-silenced and control pepper plants before and after ABA treatment **(E)**. Average leaf temperatures of *CaPRR2*-silenced and control pepper plants before and after ABA treatment **(F)**. **(G,H)** Stomatal apertures of *CaPRR2*-silenced (TRV2:*CaPRR2*) and control (TRV2:00) pepper plants in first and second leaf peels incubated in stomatal opening solution containing 0μM or 20μM ABA. Stomatal pore images of *CaPRR2*-silenced and control pepper plants. **(G)** Stomatal apertures (width/length) of *CaPRR2*-silenced and control pepper plants **(H)**.

**Figure 4:**
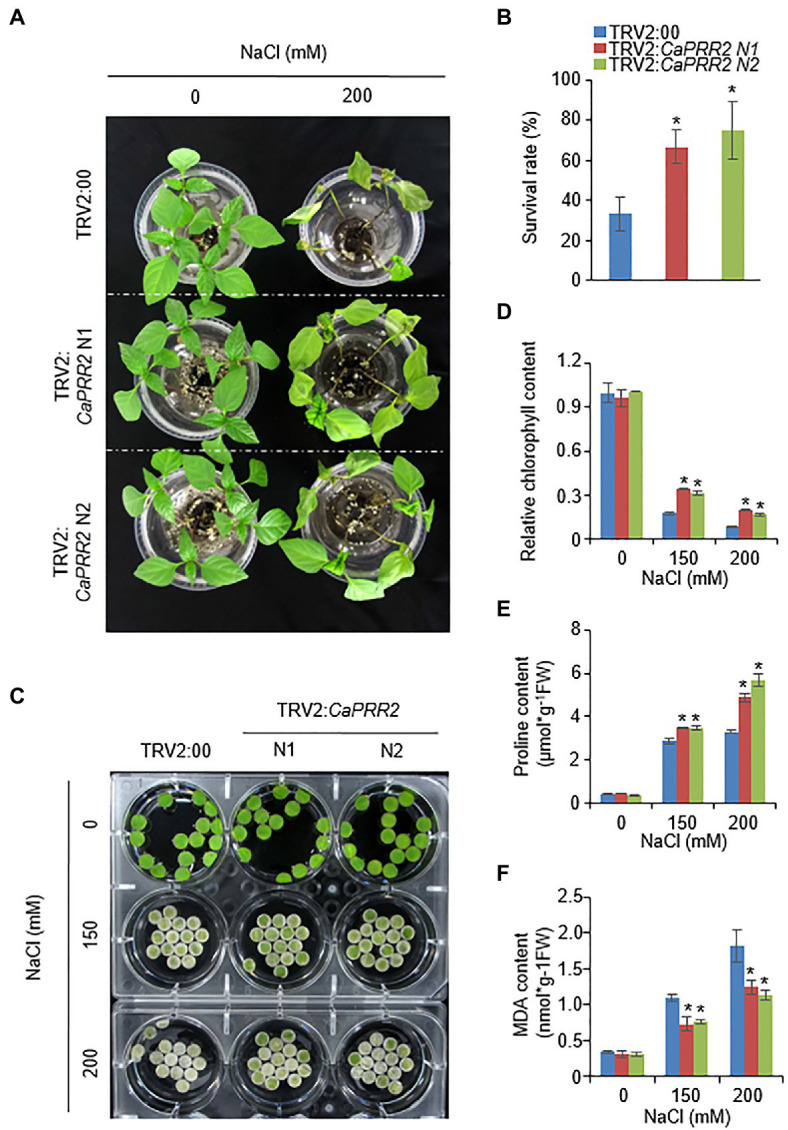
Enhanced tolerance of *CaPRR2*-silenced pepper plants to salt stress. **(A)** Salt-tolerant phenotypes of TRV2:*CaPRR2* and TRV2:00 pepper plants. Three-week-old plants of each line were hydroponically subjected to salt stress using water containing 200mM NaCl. After salt stress treatment for 4days, representative images were taken. **(B)** Survival rates of TRV2:*CaPRR2* and TRV2:00 pepper plants. **(C)** Chlorophyll contents of TRV2:*CaPRR2* and TRV2:00 plant leaf discs in various concentrations of NaCl solution. After salt stress treatment for 5days, representative images were taken. **(D)** Percentage chlorophyll contents of TRV2:*CaPRR2* and TRV2:00 plant leaf discs in NaCl solution. The chlorophyll content in non-treated TRV2:00 plants was set to 100%. **(E)** Proline contents of TRV2:*CaPRR2* and TRV2:00 plants under salt stress. **(F)** Malondialdehyde (MDA) contents of TRV2:*CaPRR2* and TRV2:00 plants exposed to salt stress.

The expression levels of *CaPRR2* varied according to different stress treatments; hence, *CaPRR2* is likely involved in abiotic stress signaling. We performed a phenotypic analysis of pepper plants using VIGS assays (Figures 3, 4). First, we generated two VIGS constructs – *CaPRR2* N1 (1,052–1,351bp) and *CaPRR2* N2 (1,361–1,660bp) – in the *CaPRR2* gene. Using RT-PCR analysis, we verified that the expression levels of *CaPRR2* were significantly lower in the *CaPRR2*-silenced pepper plants (TRV2:*CaPRR2* N1 and TRV2:*CaPRR2* N2) than in the control pepper plants (TRV2:00; Supplementary Figure S2). To examine the drought response of *CaPRR2*-silenced pepper plants, we withheld the watering of TRV2:*CaPRR2* and TRV2:00 plants for 14days and then re-watered the plants for 2days (Figure 3A). Under well-watered and drought stress conditions, we observed no phenotypic differences between both plant lines (Figure 3A, left panel). However, after re-watering for 2days, TRV2:*CaPRR2* pepper plants showed a less wilted phenotype than TRV2:00 plants (Figure 3A, right panel). The survival rates of TRV2:*CaPRR2* and TRV2:00 pepper plants were 58.3–61.1 and 30.5%, respectively (Figure 3B). We wondered whether this drought-tolerant phenotype of *CaPRR2*-silenced pepper plants is derived from alteration of water status. Since leaf relative water content is widely used as an indicator of water stress in plants, we monitored a change in the relative water content from TRV2:*CaPRR2* and TRV2:00 plants in response to drought stress. As shown in Figure 3C, TRV2:*CaPRR2* plant lines displayed significantly higher relative water content at all time points after drought treatment than TRV2:00 plants. We also evaluated the transpirational water loss by measuring the leaf fresh weights of TRV2:*CaPRR2* and TRV2:00 pepper plants. At various time points after detachment, the transpirational water loss was significantly lower in TRV2:*CaPRR2* than in TRV2:00 plants, consistent with a decrease in the relative water content (Figure 3D).

More the 99% of total water loss from leaf occurs through stomata (Kane et al., 2020). Plants close stomata in response to water deficit, and it is well-known that phytohormone ABA is involved in this process (Cutler et al., 2010). Based on these, we hypothesized that the drought-tolerant phenotype displayed by *CaPRR2*-silenced pepper plants may be caused by altered ABA sensitivity. To prove this, we measured leaf temperatures and stomatal apertures before and after ABA treatment. At 6h after ABA treatment, leaf temperatures were significantly higher in TRV2:*CaPRR2* than in TRV2:00 plants (Figures 3E,F). Moreover, at 3h after ABA treatment, stomatal apertures of TRV2:*CaPRR2* were significantly smaller than those of TRV2:00 plants (Figures 3G,H). Taken together, these results revealed that *CaPRR2* functions as a negative regulator of drought stress by regulating ABA-induced stomatal closing.

### Enhanced Tolerance of *CaPRR2*-Silenced Pepper Plants to Salt Stress

Next, we explored whether the biological role of CaPRR2 is associated with the response to salt stress. To conduct phenotypic analyses under salt stress conditions, 3-week-old TRV2:*CaPRR2* and TRV2:00 plants were subjected to salt stress by hydroponically growing them in 200mM NaCl solution. In the absence of NaCl, we observed no phenotypic differences between TRV2:*CaPRR2* and TRV2:00 plants (Figure 4A, left panel). However, when both plant lines were subjected to salt stress for 5days, TRV2:*CaPRR2* exhibited a less wilted phenotype than TRV2:00 plants (Figure 4A, right panel). The survival rates of TRV2:*CaPRR2* were 66.6–75%, whereas only 33.3% of TRV2:00 plants resumed their growth (Figure 4B). As shown in Figure 4A, salt stress triggered leaf senescence; the leaves of both plant lines treated with salt stress turned to pale yellow, compared to the non-treated plants. To evaluate this difference quantitatively, we compared the chlorophyll content between TRV2:*CaPRR2* and TRV2:00 plants using leaf discs from plants exposed to salt stress (Figure 4C). Consistent with the salt-tolerant phenotype, TRV2:*CaPRR2* plants exhibited significantly higher chlorophyll contents than TRV2:00 plants (Figure 4D). Plants resist osmotic stress by induced salt stress through production of various osmoprotectants, including proline (Nuccio et al., 1999). Measurement of proline content from TRV2:*CaPRR2* and TRV2:00 plants treated with salt stress revealed that proline content after treatment was significantly higher in TRV2:*CaPRR2* than in TRV2:00 (Figure 4E). Salt stress can induce membrane lipid peroxidation (Katsuhara et al., 2005); hence, we also measured MDA contents from TRV2:*CaPRR2* and TRV2:00 plants under salt stress conditions. MDA was highly accumulated in both plant lines by salt stress, but TRV2:*CaPRR2* had lower MDA contents than TRV2:00 (Figure 4F). These results indicate that *CaPRR2* plays a negative role in salt tolerance.

### Expression Levels of Stress-Induced Genes in *CaPRR2*-Silenced Pepper Plants Under Stress Conditions

**Figure 5:**
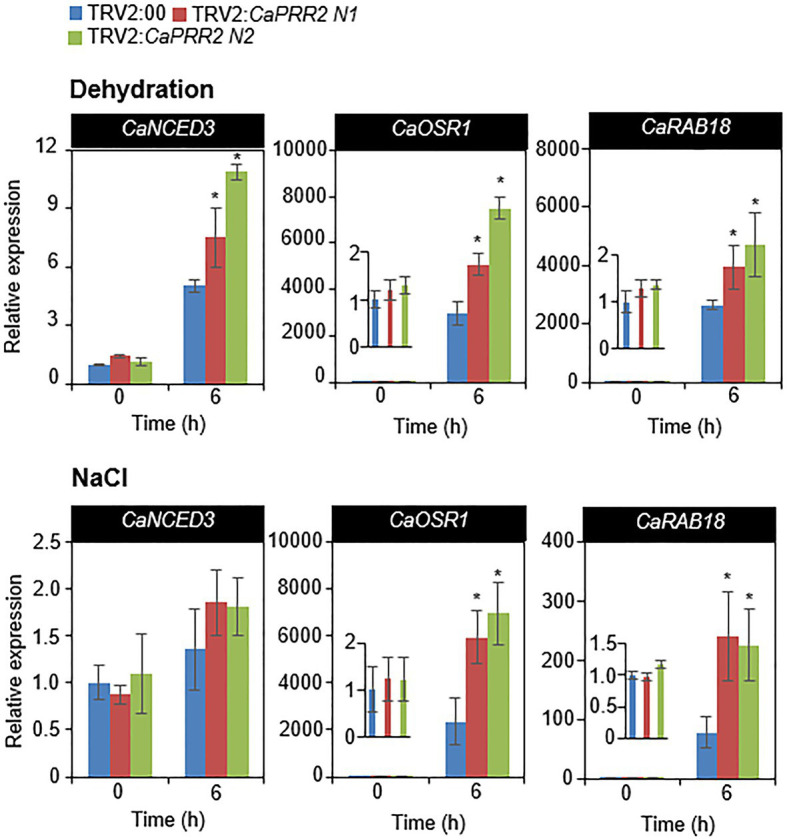
Quantitative real-time polymerase chain reaction (qRT-PCR) analysis of stress-responsive genes in the leaves of TRV2:*CaPRR2* plants. The expression levels of stress-responsive genes were analyzed in the leaves of pepper plants subjected to drought and salt stress. The relative expression levels (ΔΔ*C*T) of each gene were normalized to that of pepper *Actin1* as an internal control gene. The expression level of each gene at 0h was set to 1.0. All data represent the mean±standard error of three independent experiments. At least 16 plants per line per experiment were used. Asterisks indicate significant difference between TRV2:*CaPRR2* and TRV2:00 plants (Student’s *t* test; *p*<0.05).

Several previous studies have suggested that stress-related genes regulate osmotic tolerance (Zhang et al., 2006; Tran et al., 2007; Aubert et al., 2010). When the expression levels of stress-related genes are altered, osmotic tolerance increases or decreases. *CaPRR2* expression was negatively correlated with drought and salt tolerance. We wondered how silencing of *CaPRR2* gene affects the expression levels of stress-related genes in response to drought and salt stress. To test this, we performed qRT-PCR analysis of stress-related genes – including *CaNCED3*, *CaOSR1*, and *CaRAB18* – in the leaves of pepper plants that had been subjected to drought and salt stress (Figure 5). After drought stress treatment, *CaOSR1* and *CaRAB18* genes in TRV2:*CaPRR2* leaves showed approximately 1.7- to 2.6-fold and 1.5- to 1.8-fold increase, respectively, compared to those in TRV2:00 leaves. Similarly, salt stress significantly induced these genes; its levels were >2.5-fold higher in TRV2:*CaPRR2* than in TRV2:00. In contrast, *CaNCED3* expression was significantly induced by the drought treatment but not by the NaCl treatment. Drought stress induced expression level of *CaNCED3* to 1.5- to 2-fold in TRV2:*CaPRR2* compared to in TRV2:00. These data suggest that a reduced expression of *CaPRR2* affects the expression levels of stress-related genes, and this likely affects the osmotic stress response.

## Discussion

Plants are affected by diverse environmental stress conditions. Drought, salinity, and extreme temperatures induce osmotic stress in plant cells and limit crop yield. Plants have developed special defense mechanisms to adapt to these stress conditions. Plants manipulate their physiological and chemical properties according to the external environment. One of the well-known osmotic stress defense mechanisms is the regulation of gene expression through transcription factors (Xiong and Zhu, 2002). However, many aspects of this mechanism remain to be elucidated. In plants, PRRs are involved in a wide range of plant responses, including the regulation of circadian rhythm, fruit pigmentation and ripening, accumulation of carotenoids, and plant immunity (Nakamichi et al., 2010; Pan et al., 2013; Cheval et al., 2017). However, it is unclear whether PRRs are involved in the abiotic stress responses of plants. In the present study, we identified the pepper pseudo-response regulator CaPRR2 and revealed that CaPRR2 negatively regulates the osmotic stress response.

In phylogenetic tree analysis, CaPRR2/CA06g13040 is highly closed to APRR2 among *Arabidopsis thaliana* PRRs (Figure 1A). CA03g29830, CA10g11870, CA03g21230, CA12g12510, and CA10g05550 are putatively corresponding to APRR1, APRR3, APRR5, APRR7, and APRR9, respectively, involved in circadian rhythm (Matsushika et al., 2000; Makino et al., 2002; Nakamichi et al., 2010). Interaction of APRR2 with CML9 provides the possibility of functional involvement of APPR2 in abiotic stresses (Perochon et al., 2010). Based on these, we predicted that CaPRR2 could have a functionally similar role to APRR2. Prior to investigating functional role of CaPRR2, we analyzed organ-specific expression of *CaPRR2* in pepper plants. Expression of *CaPRR2* was highly induced in young leaf and stem in both seedling and mature stages, compared to the other organs (Figure 1B), suggesting that CaPRR2 functions in young leaf and stem. Especially, we used pepper leaves for investigating alteration of *CaPRR2* expression in response to various stress factors, including ABA, drought, H_2_O_2_, mannitol, NaCl, and low temperature (Figure 1C). *CaPRR2* showed two distinct expression patterns; ABA, drought, and mannitol decreased *CaPRR2* expression, whereas H_2_O_2_, NaCl, and low temperature increased expression. The data suggest that *CaPRR2* expression may be suppressed by cellular dehydration, probably mediated by ABA. In addition, it provides the possibility that CaPRR2 is functionally involved in abiotic stress signaling. Moreover, CaPRR2 has an MYB domain in the C-terminal region, and this domain is necessary for its localization in the nucleus (Figure 2), like APPR2 (Perochon et al., 2010). In plants, MYB transcription factors generally have DNA-binding activity and are associated with protein-protein interactions. They play an important role in biotic and abiotic stress responses. For example, MYB transcription factors regulate the synthesis and accumulation of UV-B-absorbing compounds (Jin et al., 2000; Fornale et al., 2014). Moreover, MYB transcription factors play a role in drought tolerance by affecting the expression of downstream genes in ABA-dependent and ABA-independent pathways (Roy, 2016). These findings provide the possibility that CaPRR2 may function in various environmental stress responses, especially in nucleus.

To investigate functional role of CaPRR2 in plant responses to drought and salt stress, we generated *CaPRR2*-silenced pepper plants using VIGS technique due to low transformation efficiency. In response to drought stress, *CaPRR2*-silenced pepper plants showed enhanced tolerance, compared with the control plants (Figure 3). Especially, silencing of *CaPRR2* gene was shown to be suppressed water loss through stomata, given the higher leaf relative moisture content, lower transparent water loss, and small pore size compared to the control pepper plant. Under drought stress conditions, ABA promotes stomatal closure, leading to reduced water loss (Cutler et al., 2010; Kim et al., 2010). Compared with the control plants, *CaPRR2*-silenced pepper plants displayed an ABA-sensitive phenotype characterized by significantly higher leaf temperatures and significantly smaller stomatal apertures (Figures 3E–H), suggesting that *CaPRR2* negatively regulates drought stress probably by regulating ABA-induced stomatal closing. Similar to drought, salt stress can induce osmotic stress in plants cells. High-salt levels induce ion imbalance, thereby disrupting the homeostasis of the water potential in cells (Zhu, 2001). On the salt treatment, *CaPRR2*-silenced pepper plants showed salt-tolerant phenotype than control plants (Figures 4A,B), characterized by significantly higher chlorophyll and proline contents (Figures 4D,E) and significantly lower MDA content (Figure 4F). Salt stress induces leaf senescence, leading leaf yellowing and decreased chlorophyll content, but many of these regulatory mechanisms still remained unclear (Munns and Tester, 2008; Dong et al., 2021). In salt-stressed plants, this low chlorophyll content has been considered a typical symptom of oxidative stress (Smirnoff, 1996). Measurement of MDA, a stable product of lipid peroxidation, is also considered as an indicator of oxidative damage (Del Rio et al., 2005). MDA is accumulated by salt stress and a change in its level indicates cell membrane damage and leakage under stress condition (Katsuhara et al., 2005). Furthermore, proline content is shown to have positive correlation with tolerance to abiotic stresses such as salinity (Ashraf and Foolad, 2007). Under salt stress condition, proline, most common endogenous osmolyte, is accumulated and proline functions as osmoprotectant and antioxidant, protecting cells against oxidative stress damage that causes lipid peroxidation (Hayat et al., 2012; Hasanuzzaman et al., 2020). Exogenous application of proline has been reported to improve salt stress in various plant species, including maize (Heuer, 2003; El Moukhtari et al., 2020). Based on these, we propose that *CaPRR2*-silenced pepper plants may be tolerant to salt stress by alleviating cell damage induced by oxidative stress. Taken together, *CaPRR2* negatively regulates the osmotic stress response.

The expression levels of stress-responsive genes are also related to environmental stress tolerance (Fujita et al., 2011). We measured the relative expression levels of stress-responsive genes – including *CaNCED3*, *CaOSR1*, and *CaRAB18* – in *CaPRR2*-silenced and control pepper plants after exposure to drought and salt stress. Under drought and high-salt stress conditions, the expression levels of *CaOSR1* and *CaRAB18* were significantly higher in *CaPRR2*-silenced pepper plants than in the control pepper plants (Figure 5). However, *CaNCED3* expression was significantly induced by drought but not by the high-salt treatment. Upon drought, *NCED3* expression and ABA synthesis in various plant tissues increases, leading to the activation of ABA-dependent signaling (Iuchi et al., 2001). Moreover, NCED3 positively modulates the expression of stress-responsive genes, which are associated with plant defense responses to environmental stress (Urano et al., 2009). Therefore, upregulation of *NCED3* presumably affects the defense response to drought stress. In Arabidopsis, *NCED3* gene is strongly induced by salt stress, but it is also observed in even ABA-deficient mutants, meaning that NaCl-induced *NCED3* gene is independent on ABA and dependent on NaCl (Barrero et al., 2006). Unexpectedly, salt stress triggered slight, but not statistically significant, induction of *CaNCED3* in both *CaPRR2*-silenced pepper plants than in the control pepper plants. In fact, *CaNCED3* was isolated on a basis of sequence homology with Arabidopsis *NCED3* and is not fully characterized in pepper plants. Therefore, we did not exclude the possibility that the time point used in this study is not suitable for analysis of *NCED3* gene induction by salt stress, which will be solved in further study. These data indicate that CaPRR2 directly or indirectly regulates the expression levels of these genes and functions upstream of these genes in the osmotic stress response.

In conclusion, we have demonstrated that CaPRR2 negatively regulates osmotic stress responses through biochemical and molecular changes. We were unable to identify the downstream target genes regulated by CaPRR2; hence, further studies to identify the downstream target genes controlled by CaPRR2 and to elucidate the signaling pathway involved in the CaPRR2-mediated regulation of the osmotic stress response are required. Nevertheless, our findings provide valuable insights into the plant adaptive response to osmotic stress.

## Data Availability Statement

The original contributions presented in the study are included in the article/Supplementary Material, and further inquiries can be directed to the corresponding author.

## Author Contributions

JL and CL performed the experiments and analyzed the results. SL designed the experiments and wrote the manuscript. All authors contributed to the article and approved the submitted version.

## Funding

This work was supported by a grant from “the Next-Generation BioGreen 21 Program for Agriculture and Technology Development (Project No. PJ01479801)” and the National Research Foundation of Korea (NRF) grant funded by the Korean Government (MSIT; No. 2021R1A2C2006338), Rural Development Administration, Republic of Korea.

## Conflict of Interest

The authors declare that the research was conducted in the absence of any commercial or financial relationships that could be construed as a potential conflict of interest.

## Publisher’s Note

All claims expressed in this article are solely those of the authors and do not necessarily represent those of their affiliated organizations, or those of the publisher, the editors and the reviewers. Any product that may be evaluated in this article, or claim that may be made by its manufacturer, is not guaranteed or endorsed by the publisher.

## Footnotes

^1^ http://vigs.solgenomics.net

^2^ https://web.expasy.org/compute_pi/

^3^ https://www.ebi.ac.uk/Tools/msa/clustalo/

## Supplementary Material

Supplementary Figure S1 Multiple sequence alignment analysis of the CaPRR2. Amino acid residues of the cheY-homologous receiver domain of CaPRR2 and its homologous proteins from other plant species, including *Solanum tuberosum*, *Solanum lycopersicum*, *Vitis vinifera*, *Glycine max*, *Medicago truncatula*, and *Arabidopsis thaliana*, are underlined in red, and the Myb-like DNA-binding domain of CaPRR2 and its homologous proteins from other plant species are underlined in green according to the percentage identity in ClustalW2.

Supplementary Figure S2 Reverse transcription-polymerase chain reaction (RT-PCR) analysis of *CaPRR2* expression in the leaves of TRV2:*CaPRR2* plants. The pepper *Actin1* (*CaACT1*) gene was used as an internal control.
