# Supplementary material for: Pepper Novel Pseudo Response Regulator Protein CaPRR2 Modulates Drought and High Salt Tolerance
Source: Front Plant Sci. 2021 Oct 20;12:736421. doi: 10.3389/fpls.2021.736421 (PMC8563698; doi:10.3389/fpls.2021.736421)
Supplement: Supplementary file 1 [file Table_1.PDF]

Supplemental Table S1. Sequences of primers used in this study

Primer name	Primer sequence (5'-3')
For cloning	
CaPRR2	Forward: ATGGTTTGCACTGAGAATGACTTACTG
(CA06g13040[1])	Reverse: TCAATGCGGGCGACGAGAA
w/o stop codon	Reverse: ATGCGGGCGACGAGAAC
CaPRR2 1-151	Forward: ATGGTTTGCACTGAGAATGACTTACTG
	Reverse: TTTAACCGGCTTAAGCGACTC
CaPRR2 152-291	Forward: ATGGAATCTCTTTTGTCCATGCTAGA
	Reverse: ATTAGATTCAGCAGAACGAGTACTG
CaPRR2 292-451	Forward: ATGAAAGCTTCTGGTCTCCATAGTTC
	Reverse: CTCGGGTGGTTGCCATCCA
CaPRR2 452-557	Forward: ATGAATTGGCACTGGAATCCTCAC
	Reverse: ATGCGGGCGACGAGAAC
For RT-PCR	
CaPRR2	Forward: AAGAATCTCTTTTGTCCATGCTAGA
(CA06g13040)	Reverse: AGTAGTTTCGACAGATTTTGTGTCC
CaACT1	Forward: GACGTGACCTAACTGATAACCTGAT
(CA12g08730)	Reverse: CTCTCAGCACCAATGGTAATAACTT
CaNCED3	Forward: TTAAGGATCTTAAGCGTGGTTATGT
(CA08g03620)	Reverse: AGATTAGTTCAAGAACGTGAATTGG
CaOSR1	Forward: ATGGAGGCACAACTGCACCGTC
(CA03g17780)	Reverse: GGCCCACCATGAACTTCTGCAC
CaRAB18	Forward: ATGTCGCACTACGAGAACCAATATAG
(CA02g22060)	Reverse: ATCATCCTCAGAGCTGCTGGAGC
For VIGS	
XbaI-CaPRR2 N1	Forward: TCTAGATGAAAGTAGAAGGCCTGACAA
XhoI-CaPRR2 N1	Reverse: CTCGAGTCTCGGGTGGTTGCCAT
XbaI-CaPRR2 N2	Forward: TCTAGAGGAATCCTCACTCTGGACTGTAT
XhoI-CaPRR2 N2	Reverse: CTCGAG GAGAACCGTTGATGCGTG

[1] Gene locus is from Capsicum annuum cv CM334 genome (1; release 1.55).
