# Supplementary figures and images for: Pepper Novel Pseudo Response Regulator Protein CaPRR2 Modulates Drought and High Salt Tolerance
Source: Front Plant Sci. 2021 Oct 20;12:736421. doi: 10.3389/fpls.2021.736421 (PMC8563698; doi:10.3389/fpls.2021.736421)

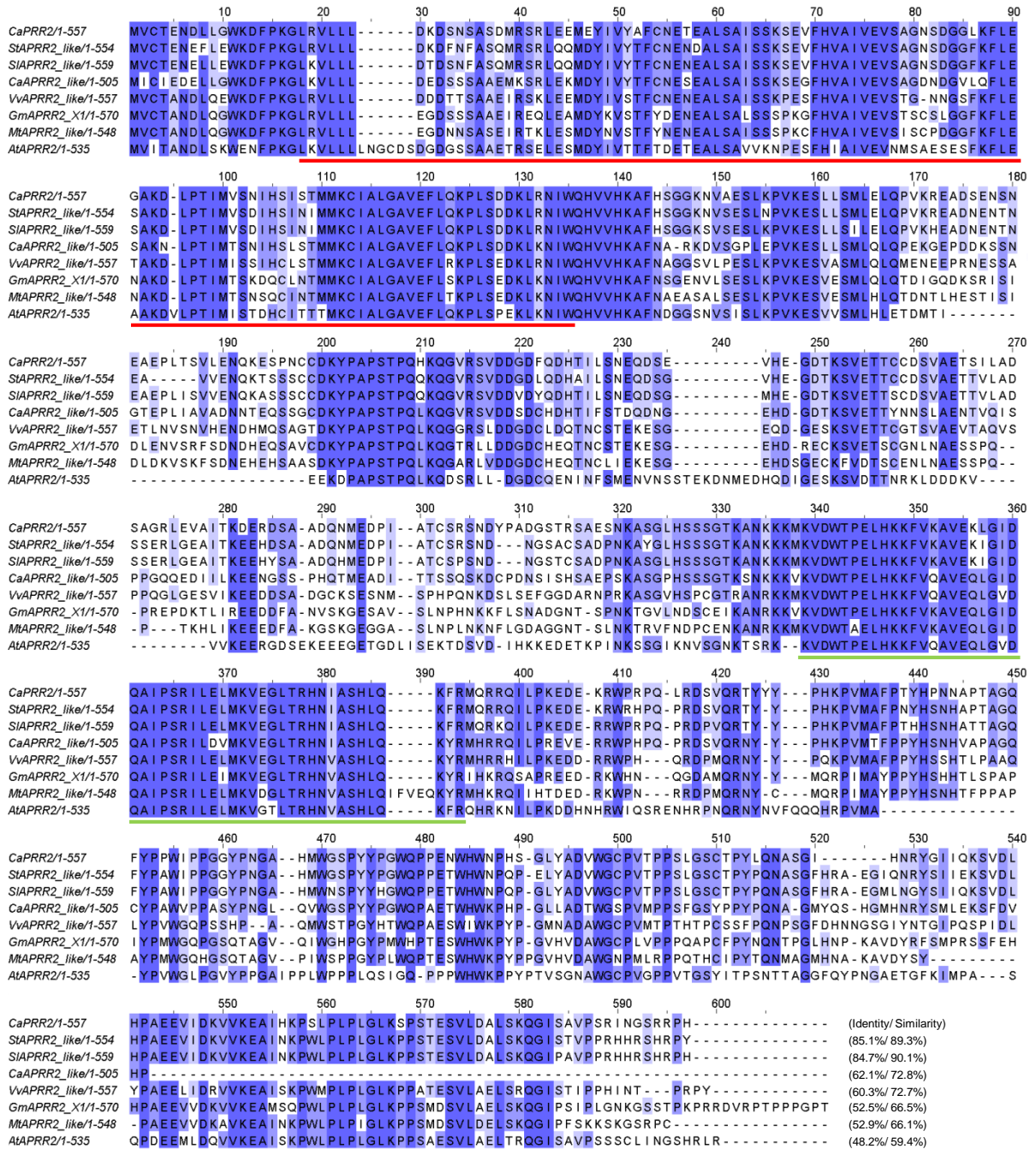

— cheY-homologous receiver domain  
— Myb-like DNA-binding domain

Supplement: Supplementary Figure S1 — Multiple sequence alignment analysis of the CaPRR2. Amino acid residues of the cheY-homologous receiver domain of CaPRR2 and its homologous proteins from other plant species, including Solanum tuberosum, Solanum lycopersicum, Vitis vinifera, Glycine max, Medicago truncatula, and Arabidopsis thaliana, are underlined in red, and the Myb-like DNA-binding domain of CaPRR2 and its homologous proteins from other plant species are underlined in green according to the percentage identity in ClustalW2. [file Image_1.PDF]

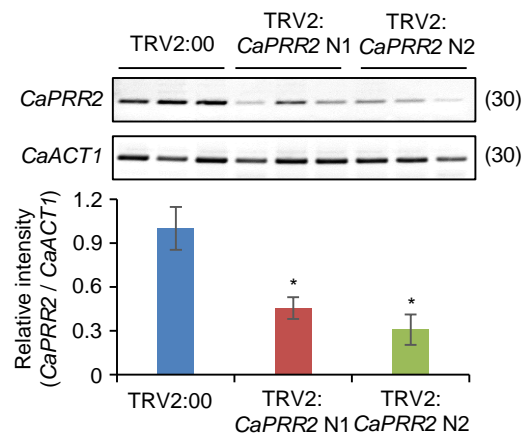

Supplement: Supplementary Figure S2 — Reverse transcription-polymerase chain reaction (RT-PCR) analysis of CaPRR2 expression in the leaves of TRV2:CaPRR2 plants. The pepper Actin1 (CaACT1) gene was used as an internal control. [file Image_2.PDF]
